# Thoracoscopic management of empyema thoracis

**DOI:** 10.4103/0972-9941.38908

**Published:** 2007

**Authors:** Michael A Wait, Daniel L Beckles, Michelle Paul, Margaret Hotze, Michael J DiMaio

**Affiliations:** Department of Cardiovascular and Thoracic Surgery, University of Texas, Southwestern Medical Center, Dallas, Texas, USA

**Keywords:** Empyema, video-assisted thoracoscopy

## Abstract

Appropriate management of empyema thoracis is dependent upon a secure diagnosis of the etiology of empyema and the phase of development. Minimal access surgery using video-assisted thoracoscopy (VATS) is one of many useful techniques in treating empyema. Complex empyema requires adjunctive treatment in addition to VATS.

## INTRODUCTION

Empyema thoracis is an infection of the pleural space that is most commonly a complication of pneumonia (parapneumonic),[1–3] but also can be a complication of primary fungal or mycobacterial infections (tuberculous), a complication of abdominal infections (sub-phrenic abscess, infected pancreas necrosis, spontaneous bacterial peritonitis) or following trauma[4] (infected retained clotted hemothorax,[5] esophageal perforation) or surgical procedures (post-resection or post-pneumonectomy empyema). Complicated parapneumonic effusion or pleural empyema develops in 10–20% of outpatients with pneumonia. Empyema is a disease process that progresses seamlessly through three recognized hallmark phases that are not sharply demarcated but represent easily identifiable points in a continuous spectrum: exudative, fibrinopurulent and fibroblastic (or organizing).

The differentiation of parapneumonic effusions, complex parapneumonic effusions and empyema has been described by Light *et al.*,[6] allowing for some degree of prognostication and appropriate treatment allocation; however, the more cumbersome classification scheme of effusions into seven different classes by Light adds essentially nothing more to the understanding of the pathogenesis or treatment of such effusions.

Treatment of empyema is dictated by an accurate diagnosis of the phase of disease progression. The most common error in treatment of empyema is failure to recognize the extent of pleural loculation and lung entrapment, resulting in persistence of inadequate futile conservative therapy.[7] If empyema has clearly progressed beyond the early exudative phase and simple thoracentesis, tube thoracostomy or catheter-based fibrinolytic therapy[8] hasn't resulted in resolution of pleural sepsis, clinical improvement or radiographic clearance within a few days, then more invasive therapy with VATS or thoracotomy is indicated. Failure to rapidly evacuate the pleural space should prompt surgical intervention and primary surgical therapy is preferred for stage II or III empyema.[8]

## DIAGNOSIS OF EMPYEMA

**Figure 1:**
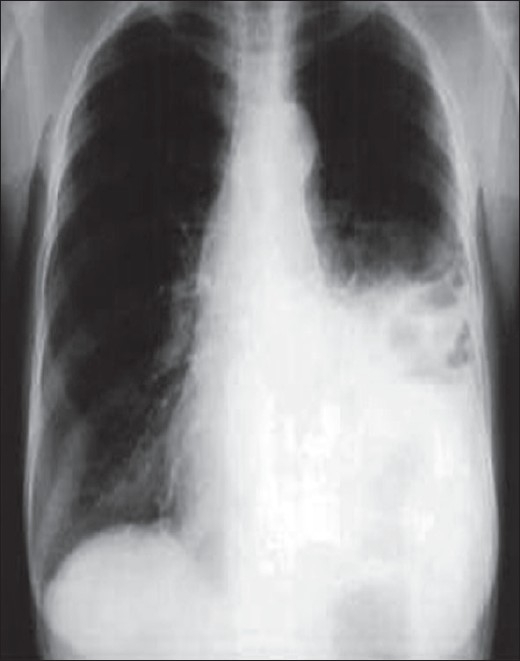
Standard AP chest X-ray depicting an elevated diaphragm with loculation of the left pleural space

Empyema thoracis has been traditionally categorized based on three clinical phases: exudative, fibrinopurulent and organizing.[1–3] The American College of Chest Physicians (ACCP) recently published an evidence-based guideline that incorporates three variables (pleural space anatomy, pleural fluid bacteriology and pleural fluid chemistry) to risk-stratify patients into four categories (1–4) that determine outcomes.[9] The initial diagnosis is made after a thorough history and physical exam to delineate the start of symptoms and prior antibiotic treatments. The standard chest X-ray is usually the first radiographic study obtained [Figure 1].

**Figure 2:**
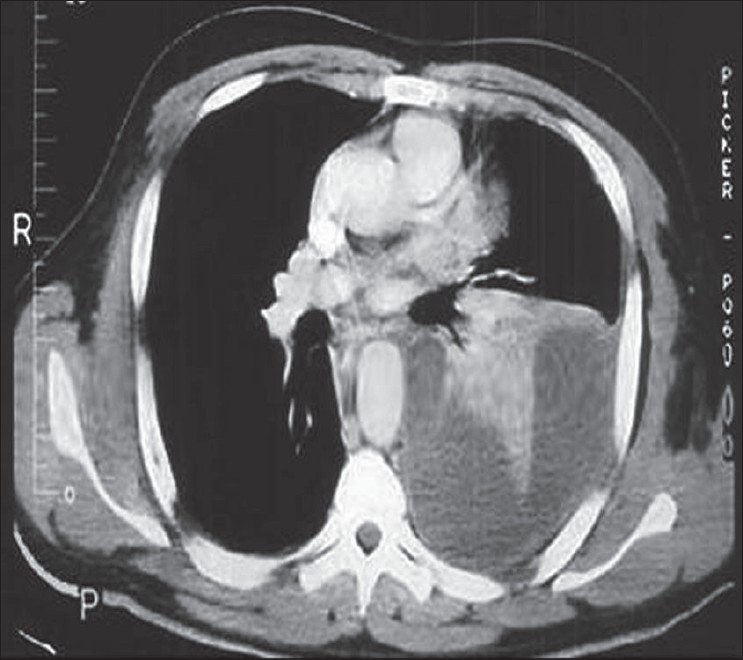
CT scan of the same patient in Figure 1, demonstrating loculation and septation of the pleural space, indicating a phase II empyema

Antibiotic therapy is initiated and a thoracentesis is performed to guide therapy (bacterial *vs* mycobacterial/fungal). A simple uncomplicated effusion/empyema can be managed with multiple thoracentesis and/or thoracostomy tube drainage with antibiotics. Daily and postprocedural X-rays are highly encouraged to detect early failures and progression of disease from simple to complicated empyema. A loculated process is suggested if after drainage there is retained fluid or air/fluid levels seen on postprocedural films and non-layering fluid on decubitus films. Ultrasound examination of the involved hemithorax has been used as an adjunct to diagnose the character of the empyema (presence or absence of septations, diaphragm immobilization, etc.), guide thoracentesis and for repeat assessment if clinical deterioration is evident. CT scans are indicated to more completely assess the anatomy of the infected pleural space, determine the presence of single *vs* multiple loculations, determine the presence or absence of a lung abscess and bronchopleural fistula and to determine adequacy of pleural drainage [Figure 2].

A bronchoscopy should be performed if there is a persistent pneumonic process, failure to improve clinically (especially from an undrained lung abscess or bronchiectasis)[10] and risk factors for bronchogenic malignancy are present

## PATHOPHYSIOLOGY

The exudative phase is frequently adequately treated by thoracentesis or closed chest tube thoracostomy alone, with appropriate antibiotics. This phase is marked by an exudative effusion which may or may not be culture positive, is frequently not loculated but free-flowing and has a leukocytic pleocytosis with polymorphonuclear neutrophilic, eosinophilc or lymphocytic cellular infiltrate. Pleural fluid pH and glucose begin to fall as leukocyte count rises and the LDH level begins to rise as well during this phase.[7] The fibrinopurulent phase is marked by increasing fibrin deposition on the visceral and parietal pleura, loculation of the pleural space and a dense purulent exudate within the pleural space, which is frequently compartmentalized. The fibroblastic phase of empyema is marked by organization of fibrin into collagenous scar, with contracture of the fibrin peel on the visceral pleura resulting in entrapment of the lung at a lower FRC (functional reserve capacity) and frequently contraction of the hemithorax.

The cytokine response to parapneumonic empyema has been referenced by Dal Nogare *et al.*[11,12] Infection stimulates the simultaneous expression of multiple pro-inflammatory cytokines, such as TNF-α (tumor necrosis factor, alpha), interleukin IL-1, IL-2, IL-6 and TGF-β (transforming growth factor beta); this last cytokine in turn stimulates over-expression of PAI-1 (plasminogen activator inhibitor, type 1) in the pleura. Expression of this cytokine causes inhibition of the normally ubiquitous pleural -derived tissue plasminogen activator, which leads to the inactivation of natural pleural fibrinolytic mechanisms. Ultimately, this results in fibrin deposition and accumulation, which promotes loculation of dense pleural exudate.

### General principles

During the fibrinopurulent, second phase of empyema, simple tube thoracostomy is inadequate and the addition of fibrinolytic therapy with streptokinase (250,000 units daily), urokinase (100,000 units daily)[7] or rTPA (retevase, 10 mg daily; Alteplase 2 mg daily[13]) frequently improves outcomes measured in terms of daily chest tube drainage and resolution of CXR findings and in some series length of hospital stay and need to progress to surgical drainage. Often, however, the addition of fibrinolytic therapy is ineffective.[14,15]

De Souza *et al.*, reported from Denver Health Medical Center in 2000 that 55% of patients in a retrospective review experienced favorable outcomes with fibrinolytic therapy added to tube drainage and that the presence of multi-loculation on CT scan was not a predictor of fibrinolytic therapy.[2] However, in our prospective randomized trial of empyema therapy at the University of Texas Southwestern Medical Center, fibrinolytic therapy was successful in only 36%.[16] Others have reported the addition of deoxyribonuclease (streptodornase) to streptokinase (Varidase)[6] or rTPA to liquefy the dense polymorphonuclear leukocyte reaction and fibrin deposition, with variable success. The addition of fibrinolytic therapy used intraoperatively with VATS has recently been reported with limited additive success.[8] Surgical debridement of the pleura with physical removal of loculation, fibrous septae, thick heavy exudate is required; historically this has been performed with thoracotomy, but in the past 15 years thoracoscopic techniques have succeeded in achieving equivalent results to open thoracotomy without the morbidity of a thoracotomy incision.

In the third phase of empyema, fibroblastic, drainage procedures alone have no merit and decortication of the visceral pleural peel is required; although this can occasionally be performed using thoracoscopic techniques, greater success at full lung decortication usually requires an open thoracotomy.

**Figure 3:**
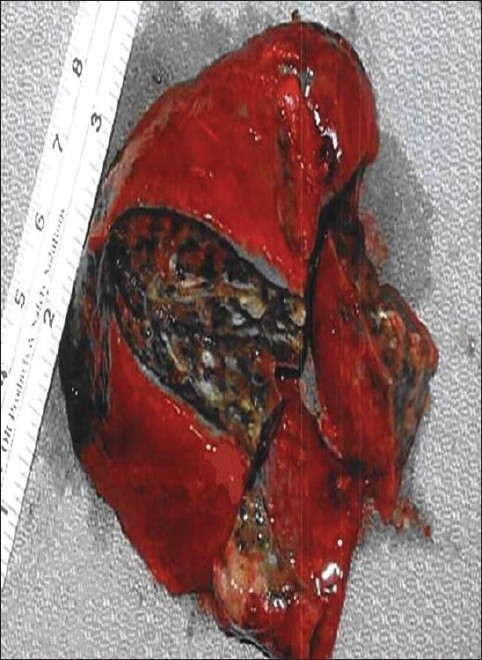
Empyemectomy specimen

One exception to this general observation is that of uniloculated putrid empyema. In this setting, a single uniloculated focus of foul, putrid thick purulence with mixed microaerophilic and anaerobic bacteria exists and is ideally treated with empyemectomy [Figure 3].

Or more commonly with rib resection (of a segment of rib approximately 4–6 cm. in Length) and placement of a large bore 5/8″ empyema tube. This empyema tube can be constructed using 5/8″ silicone tubing with multiple side holes cut out of length of the tube and connected to a standard underwater chest tube drainage system with a 5/8″–1/2″ adaptor.

In the UT Southwestern Medical Center experience of the management of empyema thoracis over the past six years, we have operated on a total of 288 patients; 81 (28%) were treated initially with VATS and 184 (64%) initially with thoracotomy; 23 (8%) were treated with rib resection and empyema tube. The VATS conversion rate to open thoracotomy was 26% (22 out of 84). The average length of stay for VATS patients was 11.6 days, VATS conversion to thoracotomy 13.5 days and open thoracotomy 18.8 days.

Not all patients with empyema thoracis require operative management, but all patients do require some sort of drainage procedure and antibiotics. In a small series of 179 patients reported from the MLK General Hospital in Los Angeles, 10% were cured with simple thoracentesis and 35% of the entire cohort remnant were cured with simple tube thoracostomy. 70% of the remaining patients were cured with thoracotomy decortication.[1] VATS was not reported in this study.

What constitutes the most appropriate treatment algorithm for a disease process which involves a dynamic spectrum has been debated in the medical literature, with most expert consensus opinion derived from registry data, retrospective reviews,[17] nonrandomized trials, historical cohort comparisons and anecdotal reports. Robust data derived from large, well done randomized clinical trials is simply lacking. 2 Cochrane Database Systematic Reviews have reported RCT comparative data on tube drainage with vs. without fibrinolytic therapy and a solitary RCT on surgical versus drainage/fibrinolytic therapy.[8,18] In a review of the Cochrane Controlled Trials Register, DARE database, MEDLINE and EMBASE databases updated as recently as February 2005, only one randomized trial regarding the surgical versus nonsurgical treatment of empyema has ever been reported.[16] Patients admitted to a university-based teaching county hospital were randomly assigned to treatment involving primary drainage with streptokinase vs. primary VATS without preliminary drainage, once the diagnosis of empyema was established by thoracentesis. The VATS group had a higher primary success rate (91% *vs* 44%, *P*<0.05) despite no increase in procedure related mortality, fewer chest-tube days (5.8 vs. 9.8, *P*=0.03), fewer ICU days (1.8 vs. 4.2, *P*=0.26), lower hospital LOS (8.7 days vs. 12.8 days, *P*=0.009). All treatment failures in the chest tube/streptokinase group were salvaged with VATS, none requiring open thoracotomy. There was one death in each group.

### Microbiology and antibiotic therapy

Most patients that develop an effusion/empyema have clinical reasons for initiating antibiotic therapy and the initial diagnostic tap for pleural fluid may yield little microbiology information with a falsely negative Gram stain. Reports of anaerobic bacteria isolated from pleural fluid have ranged from 38% to 76%.[19,20] Gram-positive cocci are the most frequently isolated organisms and the culture results are often polymicrobial consisting of anaerobic bacteria.[21] Ozol presented their retrospective review of 107 patients; 42% of patients were taking antibiotics prior to admission to the hospital. A causative agent was isolated in the pleural fluid from 40 patients and in nine blood cultures. *Staphylococcus aureus* was the most frequent bacteriological agent in 22 patients, *Streptococcus pneumoniae* in 13, anaerobes in 21 and unknown in the remaining 37 patients.[22,23] Malignancy was found in 8.4% of their patient population.

### Thoracoscopic technique

**Figure 4:**
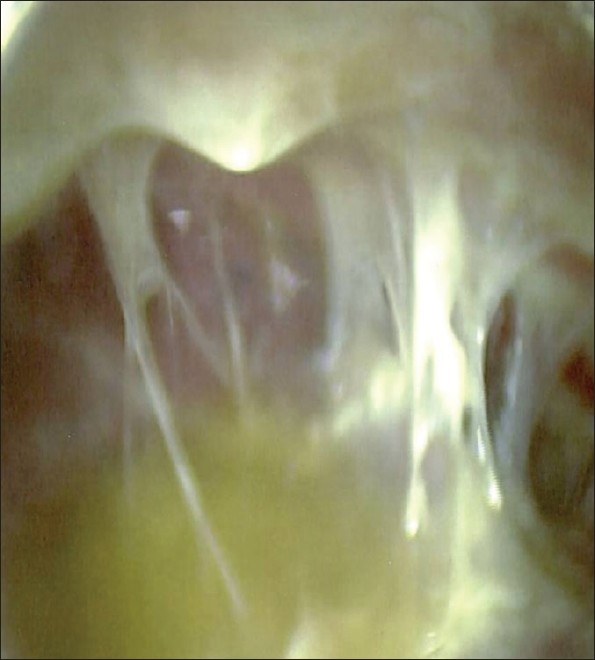
Initial thoracoscopic view of VATS decortication of a phase II (fibrinopurulent) empyema

VATS techniques provide access to the entire pleural space and offer direct, video-controlled imaging to allow for visceral and parietal pleural debridement and decortication [Figure 4].

Successful thoracoscopic treatment of empyema begins with successful anesthetic airway management. Single lung isolation with a double lumen endotracheal tube is extremely helpful, especially in cases where endobronchial aspiration is a significant risk (empyema associated with lung abscess) or has an especially morbid outcomes (post-pneumonectomy empyema with bronchopleural fistula). Intraoperative hypoxemia occurring in patients in the lateral decubitus position can be best managed by applying PEEP to the down, ventilated lung and apneic oxygenation to the non-dependant, operated lung. Apneic oxygenation can be performed by placing a small 10 Fr catheter in the lumen of the endotracheal tube that is open to atmospheric pressure and applying 5–10 liters/minute of pure oxygen flow. Although ideally suited to double lumen endotracheal tube anesthesia, apneic oxygenation can also be performed if only a single lumen tube is used. Single lumen endotracheal intubation with a bronchial blocker or selective single main bronchus intubation (especially for left sided lung isolation) is also acceptable when double lumen intubation cannot be performed for technical reasons. However, we have on many occasions of necessity performed entirely successful VATS procedures with simple endotracheal intubation without regard to bronchial isolation in critically ill patients who present to the OR in an unstable status with respiratory failure, already intubated. It is important that paralytic agents are used and the patient is in the lateral decubitus position with slight reverse Trendelenberg positioning, to allow the diaphragm to fall away from the operative field with gravity. In the morbidly obese patients with high riding diaphragms, we have found it helpful to use dependant mid-axillary port incision to provide for retraction of the diaphragm inferiorly.

Most patients referred for VATS already have at least one pleural tube in place and this tube site can be used as a camera or instrumentation port. A one-port procedure has been reported in some series[24] and should be the used for the initial evaluation of the pleural space. Typically, a total of three port incisions are utilized, one for a rigid 0-degree or 30-degree scope and the other two for instrumentation (ringed forceps, endo-Kuttners, irrigation/aspiration/suction and ultimately as sites for postoperative chest tubes). The initial camera port incision is placed in the inferior most mid-axillary level based on interpretation of the preoperative CT scan, using the xyphisternum and tip of the scapula as topical landmark references. The initial procedure is to disrupt all loculated fluid collections and to make a multiloculated empyema into a single communicating space. Liquefied exudate is aspirated and fibrin deposits are physically removed with ringed forceps. Intermittent irrigation of the pleural space with antibiotic-containing sterile water is useful to allow improved visualization of the pleural space. Once the fibrinopurulent exudate is removed, the lung is completely mobilized from the apical pleural cupola, the posterior costomediastinal gutter, the anterior pulmonomediastinal recess and the entire diaphragmatic surface of the lung, including the major and minor fissures for interlobar loculation. Finally, attempts at formal decortication, when appropriate and indicated by evidence of lung entrapment, is performed using the usual, off-the-shelf instrumentation as is used in open thoracotomy techniques. Frequently, it is appropriate to utilize a limited non-rib spreading thoracotomy (4-6 cm in length) to provide for additional standard open instrumentation for formal decortication (video-assisted thoracotomy) if significant lung entrapment is encountered. However, in more than half of cases requiring removal of a dense pleural peel, a total lung decortication will ultimately require a formal open thoracotomy to provide for total pneumonolysis, especially for tedious dissection around the diaphragm.[25] Conversion to open thoracotomy in cases with dense pleural peel from fibroblastic ingrowth should not be considered a VATS failure but as a more appropriate technique for a more advanced phase of the disease process. Fenestration of unresectable dense pleural peel or “pie-crusting” allows additional expansion of an entrapped lung if attempts at decortication cannot proceed without injuring the underlying lung.

## SPECIAL CONSIDERATIONS

### Empyema and HIV/AIDS

Pneumonia with opportunistic infections are common in HIV-infected patients as they become more profoundly immunosuppressed and transition into AIDS. Parapneumonic empyema is becoming a frequent complication in the AIDS population presenting for VATS decortication. In general, our experience has been that the VATS procedure has been equally successful in achieving relief of pleural sepsis, decortication and lung expansion as it has been in the non-AIDS population, but the empyema disease severity and the treatment outcome is determined by the CD4 (T-Helper lymphocyte) count.[26]

### Empyema associated with Bronchopleural Fistula, Necrotizing Pneumonia and Putrid Lung Abscess: Empyema in the Post-Resection Space (including Post-Pneumonectomy Empyema)

**Figure 5:**
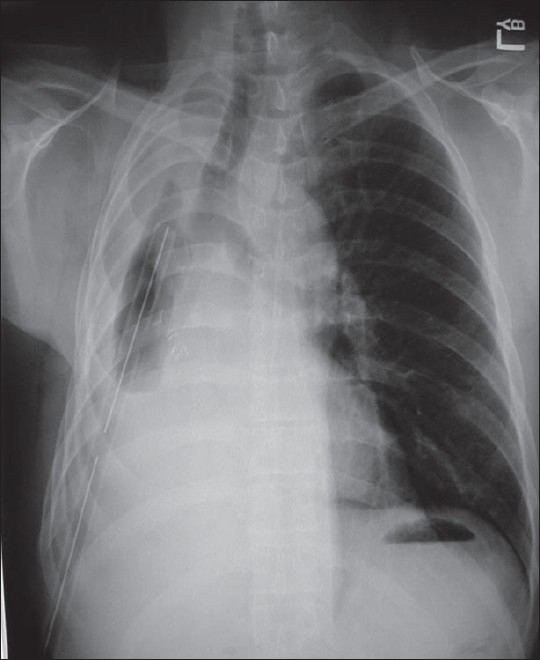
Bronchopleural fistula and empyema following right pneumonectomy for Histoplasmosis and broncholithiasis

Empyema thoracis following pneumonectomy most commonly occurs as a result of bronchopleural fistula[27] [Figure 5].

**Figure 6:**
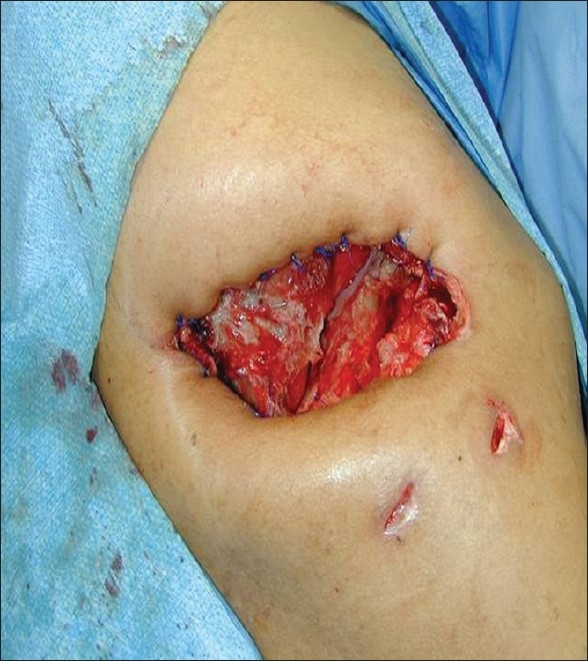
Post-resection empyema treated with Eloesser flap

**Figure 7:**
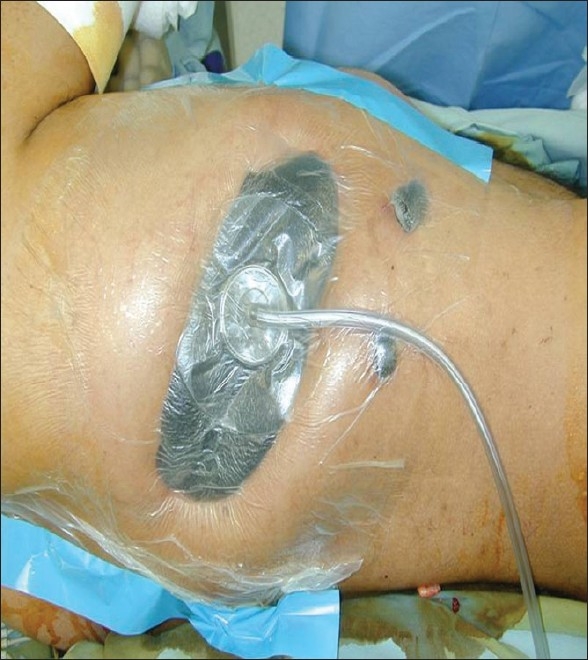
Wound vacuum placed following Eloesser flap

The rate of BPF formation following a lobectomy or pneumonectomy is approximately 4%, regardless of the surgical technique used for bronchial closure (i.e. sutured or stapled); the incidence is lowered if one uses viable tissue to buttress the bronchial stump (intercostals muscle, omentum, thymus, pericardium) and it is raised if the patient has had radiation to the bronchus. In a series of 328 consecutive extrapleural pneumonectomies performed for mesothelioma by Sugarbaker *et al.*, the incidence of BPF was 0.6% and empyema was 2.4% in a group that buttressed the short bronchial stumps with thymus and pericardium alone.[28] The initial management of PPE with BPF is VATS debridement of the pleural space and inspection of the hilum; thoracoscopic inspection of the hilar bronchovascular structures[29,30] will indicate whether a direct surgical intervention on the bronchial stump can be performed through the thoracotomy incision or if another route (trans-sternal stump closure) is more advantageous. Often the bronchial stump will require autologous tissue coverage with a latissimus, serratus or pectoralis muscle flap or omentoplasty. Frequently the postpneumonectomy space will require treatment with an open Eloesser thoracoplasty [Figures 6 and 7].

Following VATS pleural debridement and stump closure, the pleural space is irrigated and drained via a modified Clagett technique for a period of five to seven days, after which the space is filled with antibiotic containing crystalloid solution and the chest tubes are removed and sutured water-tight. Riquet[29] reported on their experience of 254 pneumonectomy patients with a PPE rate of 7% and the mean interval between surgery and PPE was 12 days (2–35 days), an antibiotic lavage regimen lasted 10 days and an 88% success rate of VATS (250,000 UI of streptokinase was instilled through chest tubes 2 hours before the surgical procedure).

**Figure 8:**
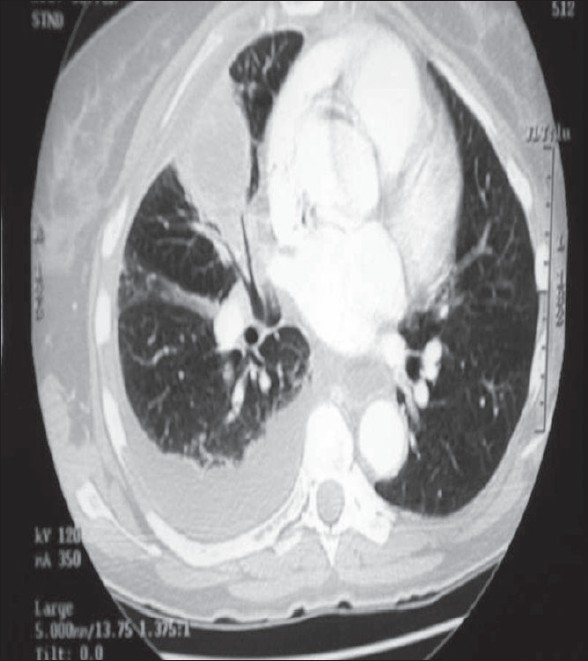
Right middle lobe lung abscess and pleural empyema

**Figure 9:**
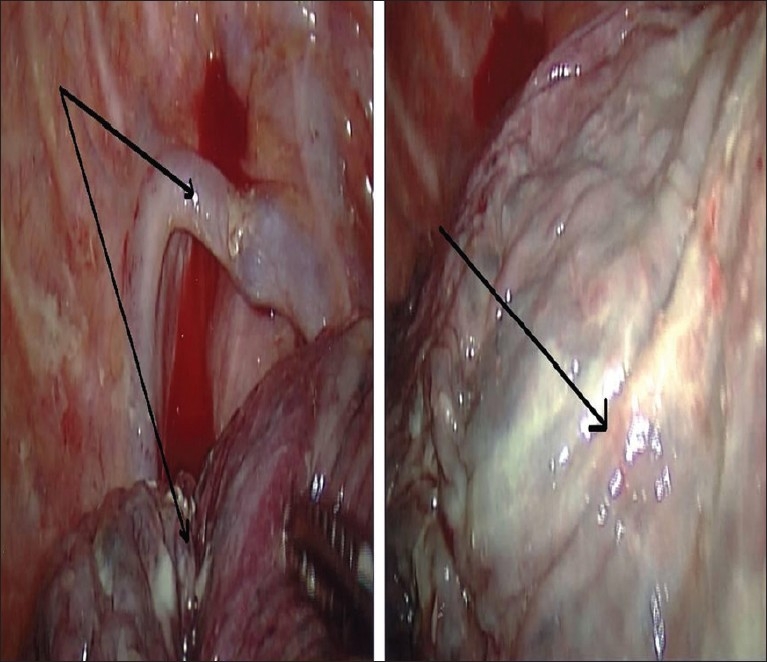
Thoracoscopic view of patient in Figure 8. There is an incidental azygous lobe (double arrows panel a) and an abscess in the right middle lobe (arrow, panel b)

Empyema can complicate a putrid lung absess, usually as a complication of erosion of the lung abscess directly into the pleural space [Figures 8 and 9].

When that occurs, if there is no associated bronchopleural fistula, the principles of surgical managaement of the empyema are unchanged. If there is a bronchopleural fistula, this has to be addressed directly either with lobar/sublobar resection or soft tissue interposition (omentum, serratus anterior, latissimus dorsi). On occasion, simple treatment of the empyema alone with decortication and drainage allows resolution of pleural sepsis and spontaneous closure of smaller bronchopleural fistulae.

### Empyema following trauma

Post-traumatic empyema as a clinical entity distinct from parapneumonic empyema has features that merit special attention. Empyema following penetrating chest and thoracoabdominal trauma was as common as following blunt trauma in one landmark study[5] (1.6% vs. 1.7%, out of a total of 5474 patients).

The microbiologic profile differs from parapneumonic empyema in that more Gram negative isolates and *Staphylococcus aureus* predominated following trauma, whereas more Gram positives (Streptococcus) and anaerobes (Fusobacterium Prevotella, Bacteroides, Peptostreptococcus) predominated following pneumonia. Post-traumatic injuries resulting in empyema commonly have the substrate of inadequately drained hemothorax,[4] heavy pleural fibrin deposition with resultant loculation, devitalizing chest wall destruction, foreign bodies (clothes, missile fragments, wadding), hollow-organ gastrointestinal contamination, hypotension and the immunosuppressive effects of transfusion, malnutrition and sepsis.

Hoth[31] reported on the pathogenesis of trauma related empyema and found little correlation between preoperative bronchoalveolar lavage/sputum cultures compared to intraoperative cultures at time of decortication excluding primary pneumonia as a cause. Presumptive antibiotic use with cefazolin was studied in a randomized trial of 224 patients randomized to antibiotics for the duration of tube thoracostomy, 24 hours of antibiotic coverage and drainage, or no antibiotics.[32] The incidence of empyema was low and the use of presumptive antibiotics did not reduce the already low risk of empyema or pneumonia.

### Empyema following esophageal perforation

Empyema is an early, first manifestation of emetogenic rupture of the esophagus (Boerhaave's syndrome), often diagnosed hours or days prior to the diagnosis of the underlying esophageal pathology. Similarly, instrumental perforation of the esophagus also quickly results in empyema if the mediastinal pleura is breached. VATS debridement and decortication of the pleural space can be used in those patients where endoscopic stent coverage of the esophageal defect has been successful,[33] obviating the need for a thoracotomy for direct repair or diversion of the esophagus.
